# Catastrophic healthcare expenditure and impoverishment in tropical deltas: evidence from the Mekong Delta region

**DOI:** 10.1186/s12939-018-0757-5

**Published:** 2018-04-27

**Authors:** Sayem Ahmed, Sylvia Szabo, Kristine Nilsen

**Affiliations:** 1 Health Economics and Financing Research Group, Health Systems and Population Studies Division, International Centre for Diarrhoeal Disease Research, Bangladesh (icddr,b), 68 Shahid Tajuddin Ahmed Sharani, Mohakhali, Dhaka, 1212 Bangladesh; 2 Department of Learning, Informatics, Management and Ethics (LIME), Karolinska Institutet, Stockholm, Sweden; 3 Department of Development and Sustainability, Asian Institute of Technology, Bangkok, Thailand; 4 Centre for Population Change, University of Southampton, Southampton, UK; 5 WorldPop, Department of Geography and Environment, University of Southampton, Southampton, UK; 6 Department of Social Statistics and Demography, University of Southampton, Southampton, UK

**Keywords:** Out-of-pocket payments, Catastrophic health expenditure, Universal health coverage, Mekong Delta, Vietnam

## Abstract

**Background:**

Universal health coverage implies that people obtain the health services they need without experiencing financial hardship. While the factors contributing to catastrophic health expenditure (CHE) among households are well understood, few studies have examined this relationship in the context of environmentally vulnerable regions, such as tropical deltas. This study aims to examine the disparities in the prevalence of CHE and impoverishment due to out-of-pocket (OOP) healthcare payments in the Mekong Delta in comparison with rest of Vietnam. It also intends to investigate the associations between economic and environmental shocks, CHE and the impoverishment from healthcare payments.

**Methods:**

Using data from the Vietnam Household Living Standards Survey 2012, the prevalence of CHE was estimated from the fraction of healthcare costs in relation to household consumption expenditure. The poverty headcount was estimated using the total household consumption expenditure considering both with and without OOP expenditure for healthcare in comparison with the national poverty-line. Simple and multiple logistic regression models were used to examine the associations between geography, health systems, environmental and demographic variables and OOP healthcare expenditure related CHE, and impoverishment respectively.

**Results:**

Both the level of OOP household healthcare expenditure and the proportion of households suffering from impoverishment as the result of such payments were higher in the Mekong Delta region compared to rest of Vietnam. Although the results from the multiple regression analysis showed that households in the Mekong Delta region were significantly less likely to suffer from CHE, they were significantly more likely to be impoverished due to OOP healthcare expenditure. While health insurance membership did not have a significant effect on either outcomes, households that faced an economic or an environmental shock in past 5 years were considerably more likely to suffer from CHE and impoverishment from OOP healthcare payments.

**Conclusions:**

The findings suggest that the financial protection capacity of health insurance schemes in Vietnam should be improved and expanded to reduce impoverishment as the result of OOP healthcare payments, particularly in the Mekong Delta region. Additional investments in disaster preparedness strategies can further help to reduce the financial burden of households in this environmentally vulnerable region.

## Background

Globally, delta regions are home to more than half a billion people and are economically important because of their relatively high agricultural production, rich biodiversity and ecosystems [1, 2]. As most tropical delta regions are located in low and middle-income countries and are prone to environmental and climate change, delta populations often suffer from multiple socio-economic and environmental vulnerabilities [3, 4]. While there is a growing literature describing the interlinkages between environmental factors and population dynamics [5–7], the research on environmental factors and health and healthcare including health expenditure in delta regions is limited. Despite varying socio-economic development of tropical deltas, people in these low-lying areas face particular health challenges resulting, amongst others, from the effects of natural hazards and creeping processes, such as soil salinization [4]. Because of relatively high population densities in these regions, environmental disasters and natural hazards are likely to affect large populations, who are often poor and have limited physical and financial access to health facilities.

In the Mekong Delta region, more specifically, the negative health effects of floods include water-borne diseases (e.g. diarrhoea), mosquito-borne diseases (e.g. malaria) and infections resulting from the contact with contaminated water [8, 9]. These health risks are, in turn, often translated into increasing out-of-pocket (OOP) expenditure on health, thus contributing to a households’ poverty and jeopardizing individual well-being [10, 11]. Expenditure on healthcare can have important effects on poverty at both micro and macro levels. At the country level, it can have a negative impact on the national poverty estimates, while at the individual level, it exacerbates household poverty, especially for those belonging to the lowest wealth quintile. Recent research from Kenya found that at the household level, 1.48 million Kenyans were pushed into poverty because of OOP healthcare payments [12]. An analysis of household survey data in Vietnam showed that between 2002 and 2010, OOP healthcare payments in Vietnam increased from US$ 4.4 to US$ 12.8. It was also found that 3.4% and 2.5% of the population become impoverished in Vietnam due to OOP healthcare payments in 2002 and 2010 respectively [11].

The large and unpredictable OOP healthcare payments can expose households to substantial financial risk and, at their most extreme, may result in impoverishment. Protecting households from such healthcare payments are desirable objectives of every health systems. Therefore, the Sustainable Development Goals (SDG), namely goal 3, include universal health coverage (UHC) as an important agenda objective [13]. The Government of Vietnam adopted a plan for achieving UHC, which aims to expand coverage of current social health insurance to at least 80% of the population and to reduce OOP healthcare expenditure to less than 40% of total healthcare spending [14]. Currently, 60% of the population in Vietnam is covered by social health insurance and households bear 47.6% of total healthcare expenditure of the country [14, 15].

In this context, the objective of this study is to examine the disparities in the prevalence of OOP health expenditure related catastrophic health expenditure (CHE) and impoverishment in the Mekong Delta region in comparison with rest of Vietnam. It also intends to investigate the associations between economic and environmental shocks, CHE and the impoverishment from healthcare payments. By conducting this analysis, this paper contributes to the literature on the determinants of CHE and impoverishment and the increasingly large body of literature on the socio-economic development of environmentally vulnerable tropical deltas [5–7].

## Methods

### Data and key variables

We used secondary data from the 2012 Vietnam Household Living Standards Survey (VHLSS). VHLSS are cross-sectional surveys regularly conducted since 1992–93 and representative at national, regional and provincial levels. The 2012 VHLSS represents the 8th round of data collection conducted through face-to-face interviews with household heads conducted by the General Statistical Office (GSO) of Vietnam. This survey employed a two-stage stratified cluster design sampling technique with a total sample of 9402 households [16, 17]. Communes were selected in the first stage and 3 enumeration areas per commune were selected in the second stage [17]. The main variables of interest in this study are the variables measuring the level of monthly consumption expenditure and OOP healthcare payments by each household in the Mekong Delta region vs. rest of Vietnam.

OOP healthcare payments refer to any payments made by households at the point they receive healthcare services (e.g. consultation fees, bed charges, diagnostic cost, medicine cost) and other related non-medical expenses (e.g. transportation cost, tips). The incidence of CHE was estimated from the fraction of OOP healthcare payments in relation to household consumption expenditure, which exceeds a certain threshold [18]. Two threshold levels were used to estimate the incidence of CHE. These are OOPs exceeding, 10% of households' total consumption expenditure [19] and 40% of household' non-subsistence expenditure [20]. The household consumption expenditure comprises both monetary and in-kind payments on all goods and services, and the monetary value of the consumption of home-made products [21]. The main explanatory variable in this study was whether the household resides in Mekong region or in another region in Vietnam. The Mekong Delta region was defined based on the administrative classification conducted by the GSO. As control variables, we included adjusted expenditure quintiles, household size, age, gender and educational attainment of the household head [11, 22]. In addition, we controlled for household location (urban vs. rural), health insurance status, if a household member were hospitalised and whether a household suffered from an economic or environmental shock in the last 5 years before the survey. As per the categorization of the GSO, these shocks included: lower incomes, job loss or underemployment, death or sickness of a household member, increased production costs, low selling prices, decreased arable land or water surface and harvest loss related to droughts, floods, and pests. Since two-stage cluster random sampling method was employed for data collection, the analyses accounted for probability sample weight. The difference in OOP healthcare expenditure, the incidence of CHE and impoverishment between Mekong Delta and rest of Vietnam were tested using independent sample t-test. Chi-square test was performed for testing association of CHE and impoverishment impact of OOP healthcare payments with socio-economic and demographic characteristics of households and other factors. For conversion from Viet Nam Dong (VND) to United States Dollar (USD), we used an exchange rate of 2012 (1 USD = 20,828 VND) [23].

### Poverty line and impoverishment

For measuring the impoverishment effect of OOP healthcare payments, the national poverty line provided by the GSO was used. The poverty headcount was estimated using total household consumption expenditure and such expenditure without OOP payments for healthcare. The gap between these two poverty headcount measurements captured the effect of OOP healthcare payments on poverty. A non-poor household was impoverished by healthcare payments when it becomes poor after paying for health services, based on the poverty line [24].

We used the GSO’s poverty line estimates based on the 2012 VHLSS data. The GSO has been using cost-of-basic-needs approach for calculation of poverty lines since the early 1990s but updated by the GSO and the World Bank following the 2010 VHLSS survey [25]. The revised consumption indices take into consideration the changing consumption patterns of Vietnamese households. The new 2010 poverty line was VND 653,000 (USD 31.4) per individual per month, which is considerably higher than the previously used benchmark [25]. For urban areas, the poverty line was VND 500,000 (USD 24.0) per person per month and for rural areas it was VND 400,000 (USD 19.2) per person per month [26]. Based on these updated thresholds, the poverty headcount was 20.7% in 2010 and 17.2% in 2012. The poverty headcount in the Mekong Delta region was slightly lower than the national average, 18.7% in 2010 and 16.2% in 2012 [27].

The Pen’s Parade graph was drawn to present the impoverishment effect of OOP healthcare payments. In this graph, household consumption was expressed as multiples of a national poverty line and presented in the vertical axis. For each household, the downward vertical bar, or “paint drop,” shows the extent to which the subtraction of OOP healthcare payments reduces consumption. If a bar crosses the poverty line, then a household is counted as poor on the basis of reduced consumption due to OOP healthcare payments [18]. We calculated number of individual fell into poverty in association with OOP healthcare expenditure by multiplying total population in 2012 with estimated additional poverty head count related to such payment.

### Econometric analysis

Simple and multiple logistic regression analyses were employed considering CHE and impoverishment as dependent variables and residence in the Mekong Delta region as the main explanatory variable of interest. The socio-economic characteristics (such as sex and education of household head, household size, expenditure quintiles, geographic location, chronic illness, health insurance status and healthcare seeking from private facilities) were included as control variables in these models. The multiple logistic regression models were specified as:

$$ \mathrm{logit}\left({\mathrm{Y}}_{\mathrm{i}}\right)={\upbeta}_0+{\upbeta}_1{\mathrm{X}}_{1\mathrm{i}}+{\upbeta}_2{\mathrm{X}}_{2\mathrm{i}}+\dots +{\upvarepsilon}_{\mathrm{i}}\dots \dots \dots .\left(\mathrm{I}\right);\kern1.5em \mathrm{i}=1,2\dots \mathrm{n} $$

In the first two model, Yi denotes incidence of CHE with a values 0 or 1 (0 = not facing CHE, 1 = facing CHE). In 3rd and 4th model, Y_i_ denotes impoverishment from OOP healthcare payments (0 = not impoverished from OOP, 1 = impoverished from OOP). For all models, β_0_is a constant and X_1i_ denotes the residence region variable (1 = Mekong region and 0 = Rest of Vietnam). The X_2i_… denote the control variables. The β_1_, β_2_…denote adjacent coefficients to the corresponding independent variables and ε_i_ represents an error term.

## Results

### Characteristics of respondents

Table 1 provides information about the characteristics of the households in the Mekong Delta region and rest of Vietnam. While male-headed households were the norm, a higher proportion of households were headed by females (28.1%) in the Mekong region, than in rest of Vietnam (24%). Households in the Mekong region also had higher proportions of heads above 60 years old and heads of Kinh ethnicity compared to rest of Vietnam. Household heads in the Mekong region also tended to have slightly lower levels of education. Household sizes were similar in both the Mekong Delta and rest of Vietnam, but households in the Mekong region contained a lower proportion of children and a higher proportion of the elderly. Overall urbanization levels were low, with about 76% and 70% of households located in rural areas in the Mekong and rest of Vietnam, respectively.

**Table 1:** Characteristics of the sample

Characteristics	Mekong Delta region (*N* = 1905)		Rest of Vietnam (*N* = 7494)		*P*-value
	%	(95% CI)	%	(95% CI)	
Sex of the household head					
Male	71.9	(69.9–73.9)	76.0	(75.1–77.0)	0.000^1)^
Female	28.1	(26.1–30.1)	24.0	(23.0–24.9)	
Age of household head					
Under 60 years	74.0	(72.0–76.0)	77.6	(76.6–78.5)	0.001^1)^
60+	26.0	(24.0–28.0)	22.4	(21.5–23.4)	
Ethnicity of household head					
Kinh	91.9	(90.6–93.1)	79.8	(78.9–80.7)	0.000^1)^
Other	8.1	(6.9–9.4)	20.2	(19.3–21.1)	
Education of the household head					
Up to primary	75.2	(73.2–77.1)	45.1	(44.0–46.2)	0.000^2)^
Secondary	21.5	(19.7–23.4)	48.1	(46.9–49.2)	
University and others	3.3	(2.5–4.1)	6.8	(6.3–7.4)	
Household size					
1–2 persons	16.6	(15.0–18.3)	19.8	(18.9–20.7)	0.005^2)^
3–4 persons	51.4	(49.1–53.6)	50.2	(49.1–51.3)	
5 persons or more	32.0	(29.9–34.1)	30.0	(28.9–31.0)	
Having elderly people in the household					
No	65.9	(63.8–68.1)	69.7	(68.6–70.7)	0.002^1)^
Yes	34.1	(31.9–36.2)	30.3	(29.3–31.4)	
Having child in the household					
No	35.6	(33.5–37.8)	41.8	(40.7–42.9)	0.000^1)^
Yes	64.4	(62.2–66.5)	58.2	(57.1–59.3)	
Observed economic/environmental shock during past 5 years					
No	73.0	(71.0–75.0)	85.6	(84.8–86.4)	0.000^1)^
Yes	27.0	(25.0–29.0)	14.4	(13.6–15.2)	
Location					
Urban	23.9	(22.0–25.9)	30.0	(28.9–31.0)	0.000^1)^
Rural	76.1	(74.1–78.0)	70.0	(69.0–71.1)	
Household with at least one health insurance enrolee					
No	17.4	(15.7–19.1)	10.8	(10.1–11.6)	0.000^1)^
Yes	82.6	(80.9–84.3)	89.2	(88.4–89.9)	
Hospitalized member					
No	19.6	(17.8–21.4)	42.3	(41.2–43.4)	0.000^1)^
Yes	80.4	(78.6–82.2)	57.7	(56.6–58.8)	
Utilized healthcare from private facility					
No	51.1	(48.8–53.3)	72.5	(71.5–73.5)	0.000^1)^
Yes	48.9	(46.7–51.2)	27.5	(26.5–28.5)	
Expenditure quintile					
Poorest	28.8	(26.7–30.8)	17.8	(16.9–18.6)	0.000^2)^
2nd	25.6	(23.6–27.5)	18.6	(17.7–19.5)	
3rd	18.2	(16.5–19.9)	20.5	(19.5–21.4)	
4th	16.2	(14.5–17.8)	21.0	(20.1–21.9)	
Richest	11.3	(9.9–12.7)	22.2	(21.3–23.1)	

^1)^Independent sample t-test of proportions ^2)^ Chi-square test

Almost twice as many households in the Mekong Delta region (27%) reported having experienced an environmental shock in the last 5 years compared to those in other regions (14.4%). There were also large differences between regions in terms of healthcare utilization. About four-fifths of households in the Mekong Delta region had a household member hospitalized in the past 12 months compared to about a third of households in rest of Vietnam. Households in the Mekong Delta region were also almost twice as likely to have used a private health facility. OOP expenditure on health was closely related to wealth and poverty. The wealth distribution was comparatively poorer in the Mekong Delta region with almost a third of households in the lowest quintile compared to less than a fifth in rest of Vietnam.

### Out-of-pocket payments

Table 2 summarises the average monthly household consumption expenditure, non-food expenditure and OOP healthcare payments in the Mekong Delta region and rest of Vietnam. As can be observed, the average monthly consumption expenditure was higher in the rest of Vietnam (VND 6266 vs. 4900) compared to the Mekong region. Conversely, mean monthly non-food consumption expenditure was slightly higher in the Mekong Delta region (VND 870 vs. 836). Finally, mean OOP healthcare expenditure was higher in the Mekong Delta region.

**Table 2:** The average monthly household consumption expenditure, non-food expenditure and out of pocket (OOP) healthcare payments in 1000 VND and USD across expenditure quintiles

Expenditure		Region				*p*-value*
		Mekong Delta region (N = 1905)		Rest of Vietnam (N = 7494)		
		In 1000 VND	In USD	In 1000 VND	In USD	
Monthly consumption expenditure	Mean	4900.4	235.3	6266.6	300.9	0.000
	Median	3911.3	187.8	5222.0	250.7	
	SD	4593.3	220.5	5037.1	241.8	
Monthly non-food expenditure	Mean	870.3	41.8	836.4	40.2	0.075
	Median	710.0	34.1	645.0	31.0	
	SD	720.2	34.6	747.4	35.9	
Monthly OOP healthcare expenditure	Mean	339.5	16.3	284.4	13.7	0.003
	Median	112.9	5.4	91.7	4.4	
	SD	876.4	42.1	685.7	32.9	

*Independent sample t-test of mean difference

### Catastrophic health expenditure

Table 3 presents the incidence of CHE by demographic and socio-economic characteristics. The incidence of CHE was higher in the Mekong Delta region compared to rest of Vietnam considering both 10% of total household expenditure and 40% of household non-food expenditure as a threshold level. Irrespective of region, households with an elderly head, Kinh ethnicity, and smaller number of members experienced greater CHE compared to their reference categories. This was also the case for households from rural areas, households that observed economic shock in last 5 years, and households which had no health insurance. These percentages according to background characteristics also tended to be higher in the Mekong Delta region compared to rest of Vietnam particularly when CHE is defined as 10% of total household expenditure.

**Table 3:** Proportion of population facing catastrophic health expenditure (CHE) in Mekong Delta regions and rest of Vietnam by demographic characteristics

Characteristics	CHE using 10% of total household expenditure as threshold level				*P*-value	CHE using 40% of household non-food expenditure as threshold level				*P*-value
	Mekong Delta region		Rest of Vietnam			Mekong Delta region		Rest of Vietnam		
	%	95% CI	%	95% CI		%	95% CI	%	95% CI	
Sex of the household head										
Male	17.3	(15.3–19.3)	11.2	(10.4–12.0)	0.000^1)^	25.7	(23.4–28.0)	23.3	(22.2–24.4)	0.000^1)^
Female	23.2	(19.6–26.8)	13.3	(11.7–14.9)		31.8	(27.8–35.7)	26.4	(24.3–28.4)	
Age of household head										
Under 60 years	16.4	(14.5–18.3)	9.3	(8.5–10.0)	0.000^1)^	23.3	(21.1–25.5)	20.1	(19.1–21.2)	0.000^1)^
60+	26.3	(22.4–30.1)	20.1	(18.1–22.0)		39.0	(34.7–43.3)	37.6	(35.3–39.9)	
Ethnicity of household head										
Kinh	19.7	(17.8–21.5)	13.4	(12.6–14.3)	0.000^1)^	28.3	(26.2–30.4)	27.0	(25.8–28.1)	0.000^1)^
Other	11.0	(6.0–15.9)	4.8	(3.7–5.9)		17.4	(11.4–23.4)	12.5	(10.9–14.2)	
Education of the household head										
Upto primary	20.5	(18.4–22.6)	12.3	(11.2–13.4)	0.000^2)^	28.6	(26.3–31.0)	23.7	(22.3–25.2)	0.004^2)^
Secondary	14.1	(10.8–17.5)	11.4	(10.3–12.4)		24.1	(20.0–28.3)	25.1	(23.7–26.5)	
University and other	15.9	(6.8–25.0)	9.9	(7.3–12.5)		20.6	(10.6–30.7)	18.7	(15.3–22.1)	
Household size										
1–2 persons	24.9	(20.2–29.7)	18.7	(16.7–20.7)	0.000^2)^	36.6	(31.3–41.9)	36.6	(34.2–39.1)	0.000^2)^
3–4 persons	15.5	(13.3–17.8)	10.1	(9.1–11.0)		22.5	(19.9–25.1)	20.4	(19.1–21.7)	
5 persons or more	21.3	(18.1–24.6)	9.7	(8.5–11.0)		30.5	(26.9–34.2)	21.9	(20.1–23.6)	
Having elderly people in the household										
No	15.3	(13.3–17.3)	9.3	(8.5–10.1)	0.000^1)^	21.5	(19.2–23.8)	19.7	(18.6–20.7)	0.000^1)^
Yes	26.0	(22.7–29.4)	17.2	(15.6–18.7)		38.8	(35.1–42.6)	34.1	(32.2–36.1)	
Having child in the household										
No	20.6	(17.6–23.7)	15.3	(14.1–16.6)	0.000^1)^	30.5	(27.0–34.0)	30.4	(28.8–32.1)	0.000^1)^
Yes	18.0	(15.9–20.2)	9.1	(8.2–9.9)		25.7	(23.2–28.1)	19.5	(18.3–20.6)	
Observed economic/environmental shock during past 5 years			0.0							
No	15.0	(13.1–16.8)	9.9	(9.1–10.6)	0.000^1)^	23.8	(21.6–26.1)	22.0	(21.0–23.0)	0.000^1)^
Yes	29.7	(25.8–33.7)	22.6	(20.1–25.1)		37.1	(32.9–41.3)	36.2	(33.3–39.0)	
Location										
Urban	16.4	(13.0–19.9)	11.9	(10.5–13.2)	0.354^1)^	22.8	(19.0–26.7)	22.3	(20.5–24.0)	0.000^1)^
Rural	19.7	(17.7–21.8)	11.6	(10.7–12.5)		28.8	(26.5–31.2)	24.8	(23.6–26.0)	
Household with at least one health insurance enrolee										
Yes	15.1	(11.2–18.9)	11.9	(9.7–14.2)	0.731^1)^	21.7	(17.2–26.1)	22.9	(20.0–25.8)	0.067^1)^
No	19.8	(17.8–21.7)	11.7	(10.9–12.4)		28.6	(26.4–30.8)	24.2	(23.2–25.2)	
Hospitalized member										
No	2.4	(0.9–4.0)	1.8	(1.3–2.3)	0.000^1)^	4.6	(2.4–6.7)	7.3	(6.4–8.2)	0.000^1)^
Yes	23.0	(20.9–25.1)	18.9	(17.8–20.1)		33.0	(30.6–35.3)	36.3	(34.9–37.8)	
Utilized healthcare from private facility										
No	15.3	(13.0–17.6)	10.2	(9.4–11.0)	0.000^1)^	22.8	(20.2–25.5)	20.5	(19.4–21.6)	0.000^1)^
Yes	22.7	(20.1–25.4)	15.7	(14.1–17.2)		32.2	(29.2–35.2)	33.4	(31.3–35.4)	
Expenditure quintile										
Poorest	19.5	(16.2–22.8)	13.3	(11.5–15.1)	0.000^2)^	27.0	(23.3–30.7)	25.0	(22.7–27.3)	0.001^2)^
2nd	18.7	(15.2–22.2)	11.3	(9.7–13.0)		24.6	(20.8–28.5)	21.5	(19.4–23.7)	
3rd	19.6	(15.4–23.8)	9.8	(8.4–11.3)		25.9	(21.3–30.6)	22.0	(19.9–24.1)	
4th	19.2	(14.8–23.6)	12.0	(10.4–13.6)		32.1	(26.9–37.4)	24.4	(22.3–26.6)	
Richest	16.7	(11.7–21.7)	12.1	(10.6–13.7)		30.2	(24.1–36.4)	26.9	(24.8–29.1)	
Total	19.0	(17.3–20.8)	11.7	(11.0–12.4)	0.000	30.2	(25.4–29.5)	24.0	(23.1–25.0)	0.002

^1)^Independent sample t-test of proportions ^2)^ Chi-square test

### Impoverishment effects

Table 4 shows the economic impoverishment effect of OOP healthcare expenditure in the Mekong Delta and rest of Vietnam. Poverty headcount from OOP healthcare spending was higher in the Mekong Delta region compared to rest of Vietnam. Poverty headcount was also higher in the Mekong Delta region. In 2012, 0.88 million people fell below poverty line (5.0% of total population) in the Mekong Delta region for high OOP spending for healthcare.

**Table 4:** Impoverishment from out-of-pocket(OOP) healthcare expenditure in Mekong Delta and rest of Vietnam

Poverty estimate	Mekong Delta region	Rest of Vietnam	*P*-value*
	% (95% CI)	% (95% CI)	
Poverty headcount	41.1 (38.9–43.3)	25.8 (24.8–26.8)	0.000
Poverty headcount associated with OOP health expenditure	5.0 (4.0–6.0)	2.3 (2.0–2.7)	0.000
Number of individual fell into poverty in association with OOP health expenditure	875,880	1,683,860	

*Two-sample t-test of proportions

**Fig. 1:**
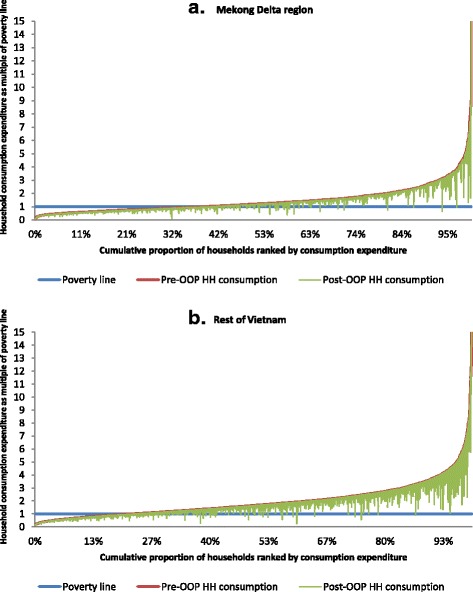
Effect of out-of-pocket payments on distribution of total household consumption in (**a**) Mekong Delta regions and (**b**) Rest of Vietnam

The impoverishment effect of OOP healthcare payments in the Mekong Delta region vs. rest of Vietnam is presented in Pen’s Parade graphs (Fig. 1 a-b). These graphs showed that OOP healthcare payments were largest at higher values in households cumulative proportion of consumption expenditure distribution. However, the households in the middle and lower half of the distribution fell below the poverty line from OOP payments for healthcare in both regions. For households which were already below the poverty line, poverty was further exacerbated for high OOP healthcare payments.

### Determinants of CHE and impoverishment

The inhabitants of the Mekong Delta were 1.766 times more likely to face CHE compared to the rest of Vietnamese using the threshold level 10% of total household expenditure (Table 5, Model 1). A similar finding (OR = 1.192; 95% CI: 1.064–1.336) was observed in simple logistic regression using 40% of household non-food expenditure as the threshold level (Table 5, Model 3). However, these results changed when multiple logistic regression models controlling for background characteristics were used. No significant association between living in the Mekong region and experiencing CHE defined as 10% of total household expenditure was observed (Table 5, Model 2). When 40% of houshold non-food expenditure was used as threshold, the direction of the association between living in the Mekong Delta region and experiencing CHE changed suggesting that Mekong Delta residents were 0.815 times less likely to face CHE compared to residents of rest of Vietnam (Table 5, Model 4). Among the control variables the significant factors for incidence of CHE, using 10% of total expenditure as a threshold, were the age of household head, ethnicity, household size, the incidence of any economic or environmental shock in past 5 years, hospitalization, private facility utilization and expenditure quintiles. On the other hand the significant controlling factors for incidence of CHE, using 40% of non-food expenditure as threshold, were the age of household head, ethnicity, education, household size, having elderly person in household, incidence of any economic or environmental shock in past 5 years, hospitalization, private facility utilization, urban and rural location, and expenditure quintiles.

**Table 5:** Determinants of catastrophic health expenditure using two threshold levels

Variable	Description	Model 1 (Dependent = CHE using 10% of total household expenditure as threshold level)		Model 2 (Dependent = CHE using 10% of total household expenditure as threshold level)		Model 3 (Dependent = CHE using 40% of household non-food expenditure as threshold level)		Model 4 (Dependent = CHE using 40% of household non-food expenditure as threshold level)	
		OR	95% CI	OR	95% CI	OR	95% CI	OR	95% CI
Mekong region	Yes (Ref = No)	1.766***	(1.544,2.021)	1.154	(0.989,1.346)	1.192**	(1.064,1.336)	0.815***	(0.714,0.931)
Sex of household head	Male (Ref = Female)			0.96	(0.826,1.118)			0.94	(0.829,1.063)
Age of household head	60+ (Ref = Under 60 years)			1.014***	(1.007,1.021)			1.015***	(1.009,1.020)
Ethnicity	Kinh (Ref = Other)			0.426***	(0.336,0.542)			0.517***	(0.436,0.613)
Education of household head	Secondary (Ref = Upto primary)			1.01	(0.870,1.172)			1.190**	(1.057,1.341)
	University and other (Ref = Upto primary)			0.97	(0.707,1.334)			0.85	(0.659,1.098)
Household size	3–4 persons(Ref = 1–2 persons)			0.660***	(0.539,0.808)			0.522***	(0.442,0.617)
	5 persons or more(Ref = 1–2 persons)			0.619***	(0.485,0.790)			0.497***	(0.408,0.607)
Having elderly people in the household	Yes (Ref = No)			1.09	(0.906,1.309)			1.235**	(1.067,1.429)
Having child in the household	Yes (Ref = No)			0.94	(0.786,1.115)			0.920	(0.797,1.052)
Observed economic/environmental shock during past 5 years	Yes (Ref = No)			2.323***	(1.997,2.702)			1.900***	(1.663,2.171)
Household with at least one health insurance enrolee	Yes (Ref = No)			1.02	(0.826,1.252)			1.180	(0.995,1.400)
Hospitalized member	Yes (Ref = No)			1.238***	(1.062,1.442)			1.545***	(1.363,1.750)
At least one member utilized private facility in last 30 days	Yes(Ref = No)			11.60***	(8.944,15.04)			6.825***	(5.873,7.931)
Location	Rural (Ref = Urban)			0.95	(0.831,1.090)			1.102	(0.985,1.234)
Expenditure quintiles	2nd (Ref = Poorest)			1.242*	(1.007,1.532)			1.199*	(1.006,1.429)
	3rd (Ref = Poorest)			1.12	(0.894,1.398)			1.251*	(1.043,1.501)
	4th (Ref = Poorest)			1.340*	(1.066,1.686)			1.549***	(1.284,1.868)
	Richest (Ref = Poorest)			1.374*	(1.072,1.760)			1.841***	(1.507,2.247)
Constant		0.132***	(0.123,0.142)	0.008***	(0.004,0.014)	0.317***	(0.300,0.334)	0.0187***	(0.012,0.030)
N		9399		9399		9399		9399	
LR chi2(19)		− 3628		− 3080		− 5252		− 4419	
Prob > chi		0.000		0.000		0.003		0.000	
Pseudo R		0.009		0.158		0.001		0.159	

Note: * p < 0.05, ** p < 0.01, *** p < 0.001

Living in the Mekong Delta was positively associated with impoverishment from health expenditure. The residents of this region were 1.356 times (95% CI: 1.005, 1.828) more likely to face impoverishment from OOP payment for healthcare (Table 6, Model 6). The households from Kinh ethnicity, with greater household size and from a rural location were less likely to face such impoverishment. Households with an older household head were 1.014 times more likely to face impoverishment, while households with a child member were 1.485 times more likely to face impoverishment. Households that faced an economic or an environmental shock in the past 5 years had a higher chance of falling below the poverty line from a health payment. If household members utilized private facilities, the household was more likely to face impoverishment from healthcare payments.

**Table 6:** Determinants of impoverishment due to out-of-pocket (OOP) healthcare expenditure

Variable	Description	Model 5 (Dependent = Poverty from OOP healthcare payments)		Model 6 (Dependent = Poverty from OOP healthcare payments)	
		OR	95% CI	OR	95% CI
Mekong Delta region	Yes (Ref = No)	2.182***	(1.692,2.816)	1.356*	(1.005,1.828)
Sex of household head	Male (Ref = Female)			0.738	(0.534,1.020)
Age of household head	60+ (Ref = Under 60 years)			1.014*	(1.000,1.027)
Ethnicity of household head	Kinh (Ref = Other)			0.490**	(0.309,0.775)
Education of household head	Secondary (Ref = Upto primary)			1.010	(0.750,1.348)
	University and other (Ref = Upto primary)			0.400	(0.121,1.340)
Household size	3–4 persons(Ref = 1–2 persons)			0.581**	(0.386,0.875)
	5 persons or more(Ref = 1–2 persons)			0.502**	(0.305,0.827)
Having elderly people in the household	Yes (Ref = No)			1.170	(0.799,1.710)
Having child in the household	Yes (Ref = No)			1.485*	(1.005,2.195)
Observed economic/environmental shock during past 5 years	Yes (Ref = No)			2.078***	(1.548,2.790)
Household with at least one health insurance enrolee	Yes (Ref = No)			1.140	(0.741,1.738)
Hospitalized member	Yes (Ref = No)			1.190	(0.860,1.650)
At least one utilized of private facility in last 30 days	Yes(Ref = No)			5.559***	(3.667,8.425)
Location	Rural (Ref = Urban)			0.810	(0.604,1.076)
Expenditure quintile	2nd (Ref = Poorest)			65.32***	(20.56,207.6)
	3rd (Ref = Poorest)			10.15***	(3.098,33.26)
	4th (Ref = Poorest)			4.533*	(1.313,15.65)
	Richest (Ref = Poorest)			1.000	(1.000,1.000)
Constant		0.0241***	(0.0207,0.0279)	0.0003***	(0.000,0.001)
N		9399		7519	
LR chi2(19)		− 1211.6		− 864.2	
Prob > chi^2^		0.000		0.000	
Pseudo R^2^		0.013		0.259	

Note: * p < 0.05, ** p < 0.01, *** p < 0.001

## Discussion

CHE and impoverishment from OOP health expenditure is a major challenge to improve health and reducing poverty [10, 12, 18, 20, 22, 28, 29]. Van Doorslaer et al. (2007), using data from VHLSS 2000, found that 15.11% and 5.97% of households in Vietnam faced CHE considering 10% of total health expenditure and 40% of non-food expenditure respectively as thresholds [10]. In another multi-country analysis, van Doorslaer et al., (2006) observed that in 11 Asian countries, 2.7% of the population under study (78 million people) fell below the poverty line due to OOP payments for healthcare [22]. The Mekong Delta region faces particular environmental and health challenges which may impact health expenditure. For example, Nguyen et al. (2017) showed that a high burden of contagious diseases was, at least partially due to environmental and climatic factors in Mekong region [30]. Unlike previous studies, this paper aims at examining the level of OOP healthcare expenditure and CHE and assessing the determinants of health expenditure and impoverishment due to such payments in the Mekong Delta region compared to rest of Vietnam. In order to achieve its first objective, the paper used standard OOP healthcare payments and CHE definitions and measurement; in order to achieve its second objective, the study considered logistic regression models.

The results showed that the level of households' CHE in the Mekong Delta region tended to be higher compared to rest of Vietnam (aggregated data) and that impoverishment effects from health expenditure in the Mekong Delta were stronger. In this region, poverty headcount due to OOP healthcare expenditure was estimated at 5.0% compared to 2.3% in rest of Vietnam. Surprisingly, results from the multiple regression models showed either no association or a negative association between living in the Mekong region and CHE. However, households in the Mekong region were much more likely to be impoverished as a result of healthcare payments compared to households in rest of Vietnam. Impoverishment from health expenditure suggested that health insurance coverage was either poor and/or did not offer sufficient financial protection [31]. Although health insurance coverage in Vietnam is high, it is not universal and this study showed that coverage levels in the Mekong Delta region were about 6.6% lower compared to the rest of the country. These finding are supported by previous studies, which found that people are paying high OOP for healthcare and facing CHE due to lower coverage of social health insurance for the population in Vietnam [9, 14]. Nevertheless, expansion of existing insurance models may not be sufficient since our results suggested that health insurance membership did not significantly reduce the likelihood of experiencing CHE or provide sufficient protection against impoverishment for OOP healthcare payments. In a recent paper, Kien et al. found that the social health insurance coverage was not effective enough to protect household from the CHE and impoverishment and he argued that the consumption of prescribed drugs that are not covered by the insurance could be a reason for this [32]. Another study showed that few drugs were prescribed (40.8%) by doctors from the essential medicine list, which were covered by social health insurance [33].

Environmental shocks experienced by households had a consistently positive effect on both CHE irrespective of threshold and on impoverishment due to healthcare payments. These findings were in line with existing evidence, which showed a negative effect of environmental shocks beyond direct income loss [34].

Among demographic and socio-economic background characteristics, having a dependent person in the household tended to increase the likelihood of either CHE or OOP healthcare payments related impoverishment. Minh et al. found that households which encountered CHE and/or were pushed into poverty for high OOP healthcare payments during the period 2002 and 2010 were more common among the households who had more elderly people and those located in rural areas [11]. It was observed that households belonging to lower expenditure quintiles were less likely to face CHE. This might reflect the inability of the poor to divert resources from other basic needs which hinder this population from seeking care when needed and to rely on self-medication and consequently less OOP healthcare payments [10, 34, 35]. In the current context of increasing public-private mix in service provision in Vietnam, another reason could be the utilization of expensive private facilities by rich people [34, 36]. Minh et al., observed a similar association between expenditure quintiles and CHE in a trend analysis of VHLSS from 2002 to 2010 using multiple regression analysis [11]. Impoverishment related to OOP healthcare payments was higher in 2nd, 3rd^,^ and 4th expenditure quintiles compared to 1st expenditure quintile. This finding was in-line with the Minh et al. [11]. Since people in the 1st expenditure quintile were already living below poverty line, fewer people in this group were at risk of falling below the poverty line due to the OOP healthcare payments. However, this could further exacerbated their poverty condition as shown in Fig. 1. Due to high OOP healthcare payments, the people from 2nd, 3rd and 4th expenditure quintiles were falling below the poverty line as found by another study [11]. However, Kien et al. observed a higher incidence of CHE and impoverishment among poor groups compared to the rich in a relatively small study (sample size = 492 slum and 528 non-slum households) in urban Hanoi, Vietnam [32]. A recent study observed that the incidence of impoverishment was decreasing with time in Vietnam [37].

One of the important determinants of CHE and impoverishment was at least one visit to a private facility in the previous thirty days. In Vietnam, private healthcare is paid for either by the patients directly or by their social health insurance or private health insurance scheme [38]. Currently, private health facilities comprise 4.2% of total hospital beds in Vietnam. Most of these facilities have less than 100 beds and located in cities and towns and offer diverse healthcare services [39]. Earlier studies recommended an increase in the number of private facilities to improve access to healthcare in Vietnam, however, unless sufficiently regulated, this can lead to increased health expenditure and might have adverse impacts on the financial health protection of households [40].

While our study contributed to the existing body of literature, it has several limitations. Use of cross-sectional data was one of the major limitations of this study. Ideally, longitudinal data would be used to estimate the causal effect of impoverishment resulting from OOP payments for healthcare. The multiple regression analysis showed associations between CHE and impoverishment impact of OOP healthcare expenditure and other factors and these cannot be interpreted as causal relationships. Another limitation was the potential for recall bias, as expenditure data were based on a self-reported questionnaire. Therefore, the estimates in this study should be interpreted taking these into consideration. However, to our knowledge this is the first study in Vietnam, showing the disparities in the impact of high OOP healthcare payments in the environmentally vulnerable Mekong Delta region compared to rest of Vietnam. Another important finding of this study was the association between climate/environmental factors and CHE and impoverishment.

## Conclusion

This study aimed to investigate the disparities in the prevalence of CHE and OOP healthcare payments-related impoverishment in the Mekong Delta region in comparison with the rest of Vietnam. It also aimed to examine the associations between environmental and economic shocks and households’ impoverishments effects. The results of our analysis showed that in the Mekong Delta region, households were significantly more likely to suffer OOP healthcare expenditure related impoverishment. In addition, the analysis showed that households that endured either economic and/or environmental shock during the past 5 years were more likely to suffer from CHE and impoverishment from OOP healthcare payments.

The results from the multiple regression analysis suggested that in the Mekong Delta region, health insurance did not offer sufficient protection against CHE and impoverishment. Priority should, therefore, be given to increasing financial protection capability of the social health insurance. Additional research should be conducted to identify why health insurance appears not to offer the intended financial protection. The findings of this study also imply that the impacts of environmental and climate change on livelihoods and public health in this region were likely to worsen households’ burden of OOP healthcare payments. Strengthening health systems in climate change hotspots, such as the Mekong Delta region, [36, 40] will, therefore, be critical. This must involve not only investing in health insurance schemes, in particular for the poorest, but also focus on initiatives to improve reduction and management of natural hazards and disasters. Climate adaptation and policy interventions which lead to a reduction of economic and/or environmental shocks and improve households’ coping strategies would be an effective measure to reduce OOP healthcare payments.

## Abbreviations

CHE: Catastrophic health expenditure
CI: Confidence interval
GSO: General Statistical Office
OOP: Out-of-pocket
SDG: Sustainable Development Goals
UHC: Universal health coverage
VHLSS: Vietnam household living standards survey
VND: Vietnamese dong
